# Supplementary material for: Clinical Outcomes with Targeted Temperature Management (TTM) in Comatose Out-of-Hospital Cardiac Arrest Patients—A Retrospective Cohort Study
Source: J Clin Med. 2022 Mar 24;11(7):1786. doi: 10.3390/jcm11071786 (PMC8999846; doi:10.3390/jcm11071786)
Supplement: Supplementary file 1 [file jcm-11-01786-s001.zip › jcm-1653575-supplementary.pdf]

SUPPLEMENTARY TABLES

Supplementary Table S1 Patient and arrest characteristics non-survivors versus survivors at day 90

Characteristics	All TTM patients	90-day mortality		p-value	Missing (n)
		Non-survivors	Survivors		
	n=798	n=322	n=476		
**Demographic**					
Age, *median [IQR]*	63.8 [53.8 - 72.2]	67.9 [59.0 - 76.5]	60.8 [50.5 - 68.5]	<0.001	0
Female, *n (%)*	191 (23.9)	87 (27.0)	104 (21.8)	0.09	0
**Medical history**					
Hypertension	283 (37.9)	123 (42.1)	160 (35.2)	0.06	52
Hypercholesterolemia	198 (26.6)	81 (27.8)	117 (25.8)	0.53	53
Diabetes Mellitus	152 (19.8)	88 (29.2)	63 (13.7)	<0.001	36
Family history of CVD	115 (18.8)	25 (11.5)	90 (22.8)	0.001	186
Smoking	215 (32.7)	92 (36.5)	123 (30.3)	0.10	140
Peripheral vascular disease	66 (8.7)	36 (12.0)	30 (6.5)	0.008	38
Previous myocardial infarction	175 (22.0)	77 (24.1)	98 (20.6)	0.24	2
Chronic heart failure	89 (11.2)	49 (15.4)	40 (8.4)	0.002	3
Previous PCI	116 (14.6)	45 (14.1)	71 (14.9)	0.73	3
Previous CABG	54 (6.8)	24 (7.5)	30 (6.3)	0.52	3
Previous ICD implantation	21 (2.6)	9 (2.8)	12 (2.5)	0.81	2
Previous pacemaker implantation	19 (2.4)	12 (3.7)	7 (1.5)	0.04	2
Previous TIA or stroke	64 (8.0)	38 (11.8)	26 (5.5)	0.001	1
Pulmonary embolism	11 (1.4)	6 (1.9)	5 (1.1)	0.33	2
**Arrest characteristics, *n (%)***					
Location of arrest				0.001	0
Home	440 (55.1)	200 (62.1)	240 (50.4)		
Public	358 (44.9)	122 (37.9)	236 (49.6)		
Witnessed arrest	592 (76.4)	225 (72.3)	367 (79.1)	0.03	23
Bystander CPR	504 (65.2)	173 (55.6)	331 (71.6)	<0.001	25
Estimated time to CPR				<0.001	127
0-5 minutes	515 (76.8)	175 (66.8)	340 (83.1)		

6-10 minutes	125 (18.6)	64 (24.4)	61 (14.9)		
11-20 minutes	26 (3.9)	19 (7.3)	7 (1.7)		
>20 minutes	5 (0.7)	4 (1.5)	1 (0.2)		
AED defibrillation	320 (40.3)	84 (26.3)	236 (49.7)	<0.001	3
Shockable initial rhythm	596 (78.1)	186 (60.0)	410 (90.5)	<0.001	35
Defibrillation by EMS	523 (65.6)	192 (59.8)	331 (69.5)	0.005	1
Time to ROSC				<0.001	149
0-5 minutes	32 (5.1)	11 (4.1)	22 (5.8)		
6-10 minutes	116 (17.9)	32 (11.8)	84 (22.2)		
11-20 minutes	297 (45.8)	103 (38.0)	194 (51.3)		
>20 minutes	203 (31.3)	125 (46.1)	78 (20.6)		
Pre-hospital intubation	509 (63.8)	234 (72.07	275 (57.8)	<0.001	0
Primary cardiac cause	693 (92.0)	255 (85.3)	438 (96.5)	<0.001	45

Note: Descriptive statistics of the sample comparing the non-survivors and survivors at day 90. IQR: Interquartile Range, CVD: Cardio-Vascular Disease, CABG: Coronary-Artery Bypass Grafting, PCI: Percutaneous Coronary Intervention TIA: Transient Ischemic Attack, ICD: Implantable Cardioverter Defibrillator, CPR: Cardiopulmonary Resuscitation, AED: Automatic External Defibrillator, EMS: Emergency Medical Service. ROSC: Return Of Spontaneous Circulation.

Supplementary Table S2 Post arrest care characteristics non-survivors versus survivors at day 90

Characteristics	All TTM patients	90-day mortality		p-value	Missing (n)
		Non-survivors	Survivors		
	n=798	n=322	n=476		
Lowest temperature during TTM, *℃ [IQR]*	33.0 [32.7 - 34.2]	33.0 [32.5 - 34.4]	33.0 [32.7 - 34.1]	0.45	117
Bradycardia during TTM*, n (%)*	389 (55.5)	128 (49.0)	261 (59.3)	0.008	49
Lowest MAP during TTM*, mmHg [IQR]*	57 [52 - 61]	56 [48 - 59]	58 [54 -62]	<0.001	51
Inotropics/vasoactive drugs*, n (%)*	737 (98.4)	291 (97.7)	440 (98.9)	0.24	49
Mechanical circulatory support, *n (%)*				0.49	0
IABP	94 (11.8)	42 (13.0)	52 (10.9)		
Impella	1 (0.1)	0 (0.0)	1 (0.2)		
ECMO	7 (0.9)	4 (1.2)	3 (0.6)		
CVVH, *n (%)*	37 (5.0)	21 (6.9)	16 (3.6)	0.07	51

Note: Descriptive statistics of the sample comparing the non-survivors and survivors at day 90. IQR: Interquartile Range, TTM: Targeted Temperature Management, IABP: Inta-Aortic Balloon Pump, ECMO: Extra Corporal Membrane Oxygenation, CVVH: Continues Veno-Venous Hemofiltration.

Supplementary Table S3 Multivariate analysis 90-days mortality, favorable neurological outcome, CPC score and ICU length of stay

	**Cox regression analysis for 90-day mortality after ICU admission**	**Binary logistic regression for favorable neurological outcome**	**Linear regression for CPC score**	**Linear regression for ICU LOS**
	(model 1)	(model 2)	(model 3)	(model 4)
TTM strategy (Ref is TTM36)	0.877 (0.677 - 1.135)	1.330 (0.941 - 1.879)	-0.128 (-0.421 - 0.166)	1.180 (0.222 - 2.138)
Hypercholesterolemia	1.081 (0.833 - 1.403)	0.819 (0.583 - 1.149)	0.082 (-0.206 - 0.369)	-0.250 (-1.178 - 0.679)
Previous ICD implantation	1.409 (0.721 - 2.756)	0.785 (0.304 - 2.027)	0.350 (-0.464 - 1.165)	0.234 (-2.648 - 3.116)
Witnessed arrest	0.744 (0.574 - 0.965)	1.347 (0.947 - 1.915)	-0.203 (-0.503 - 0.097)	0.388 (-0.592 - 1.368)

Note: Estimates are Hazard Ratios (HR), Odds Ratios (OR), and beta's, and 95% Confidence Interval in parentheses (95% CI), derived from a Cox regression analysis (model 1), Binary logistic regression analysis (model 2) and a linear regression analysis (model 3 & 4), respectively. CPC: Cerebral Performance Score, ICU: Intensive Care Unit, LOS: Length Of Stay, ICD: Implantable Cardioverter Defibrillator.

Supplementary Table S4 Subgroup analysis in patients with lowest temperature available, 90-days mortality, favorable neurological outcome, CPC score and ICU length of stay

	**Cox regression analysis for 90-day mortality after ICU admission**	**Binary logistic regression for favorable neurological outcome**	**Linear regression for CPC score**	**Linear regression for ICU LOS**
	(model 1)	(model 2)	(model 3)	(model 4)
TTM strategy (Ref is TTM36)	0.871 (0.662 - 1.146)	1.342 (0.926 - 1.946)	-0.124 (-0.443 - 0.194)	1.104 (0.121 - 2.087)
Hypercholesterolemia	1.117 (0.848 - 1.471)	0.797 (0.554 - 1.147)	0.127 (-0.185 - 0.438)	-0.524 (-1.473 - 0.424)
Previous ICD implantation	1.195 (0.561 - 2.546)	0.919 (0.326 - 2.593)	0.156 (-0.738 - 1.051)	-0.269 (-3.202 - 2.665)
Witnessed arrest	0.749 (0.569 - 0.987)	1.357 (0.931 - 1.977)	-0.223 (-0.546 - 0.100)	0.371 (-0.624 - 1.366)

Note: Estimates are Hazard Ratios (HR), Odds Ratios (OR), and beta's, and 95% Confidence Interval in parentheses (95% CI), derived from a Cox regression analysis (model 1), Binary logistic regression analysis (model 2) and a linear regression analysis (model 3 & 4), respectively. CPC: Cerebral Performance Score, ICU: Intensive Care Unit, LOS: Length Of Stay, ICD: Implantable Cardioverter Defibrillator.
